# Dosing parameters for the effects of high-frequency transcranial magnetic stimulation on smoking cessation: study protocol for a randomized factorial sham-controlled clinical trial

**DOI:** 10.1186/s40359-020-00403-7

**Published:** 2020-05-01

**Authors:** Ellen Carl, Amylynn Liskiewicz, Cheryl Rivard, Ronald Alberico, Ahmed Belal, Martin C. Mahoney, Amanda J. Quisenberry, Warren K. Bickel, Christine E. Sheffer

**Affiliations:** 1 Roswell Park Comprehensive Cancer Center, Buffalo, NY 14203 USA; 2 Fralin Biomedical Research Institute at Virginia Tech Carilion, Roanoke, VA 24016 USA

**Keywords:** Smoking cessation, Tobacco dependence, Transcranial magnetic stimulation, Relapse prevention, Brain stimulation, Neuromodulation, Delay discounting, Dorsolateral prefrontal cortex

## Abstract

**Background:**

Despite the considerable success of comprehensive tobacco control efforts, tobacco use remains one of the greatest preventable causes of death and disease today. Over half of all smokers in the US make quit attempts every year, but over 90% relapse within 12 months, choosing the immediate reinforcement of smoking over the long-term benefits of quitting. Conceptual and empirical evidence supports continued investigation of high frequency repetitive transcranial magnetic stimulation (rTMS) of the left dorsolateral prefrontal cortex in reducing relapse and decreasing cigarette consumption. While this evidence is compelling, an optimal dosing strategy must be determined before a long-term efficacy trial can be conducted. The goal of this study is to determine a dosing strategy for 20 Hz rTMS that will produce the best long-term abstinence outcomes with the fewest undesirable effects.

**Methods:**

This is a fully crossed, double-blinded, sham-controlled, 3x2x2 randomized factorial study. The three factors are duration (stimulation days: 8, 12, and 16); intensity (900 or 1800 pulses per day); and sham control. Participants (*n* = 258) will consist of adults (18–65) who are motivated to quit smoking cigarettes and who will be followed for 6 months post-quit. Outcomes include latency to relapse, point prevalence abstinence rates, delay discounting rates, cognitive-behavioral skills acquisition, and multiple measures of potential undesirable effects that impact participant compliance.

**Discussion:**

This study integrates existing theoretical concepts and methodologies from neuropsychology, behavioral economics, brain stimulation, clinical psychology, and the evidence-based treatment of tobacco dependence in the development of a promising and innovative approach to treat tobacco dependence. This study will establish an optimal dosing regimen for efficacy testing. Findings are expected to have a significant influence on advancing this approach as well as informing future research on clinical approaches that combine rTMS with other evidence-based treatments for tobacco dependence and perhaps other addictions.

**Trial registration:**

Clinical Trials NCT03865472 (retrospectively registered). The first participant was fully enrolled on November 26, 2018. Registration was posted on March 7, 2019.

## Background

Despite the considerable success of comprehensive tobacco control efforts, tobacco use remains one of the greatest preventable causes of death and disease today [1]. In the US alone, cigarette smoking causes > 480,000 deaths annually including a third of all cancer deaths, and 87% of all lung cancer deaths [1, 2]. Most smokers want to quit and over half make a quit attempt every year, but over 90% choose the immediate reinforcement of smoking over the significant long-term benefits of quitting [3–7] within 12 months. This conundrum of relapse to smoking despite considerable efforts among smokers to quit continues to be one of the most significant public health challenges today [1, 2].

Smoking cessation involves repeatedly choosing between the known, immediate, and reinforcing experience of smoking and a variety of dynamic choice options from which the smoker repeatedly chooses throughout the day in the context of continually fluctuating neurobiological, environmental, and cognitive influences [8–10]. Executive functions such as self-control are essential to managing the repetitive choice process if one is to attain long-term abstinence [11–13]. The dorsolateral prefrontal cortex (dlPFC), a functional node in the prefrontal cortex (PFC), has a significant role in self-control in general and the controlled response inhibition associated with managing the desire to smoke, called incentive salience [14–19]. Executive function neural processing deficits among smokers parallel decreased activity in the PFC [20] and compromise smokers’ ability to manage incentive salience and maintain the choice to quit smoking [21, 22]. Moreover, the absence of nicotine during a quit attempt amplifies executive function neural processing deficits, increases incentive salience, and is likely to contribute to the choice to smoke, even if it means relapse [23, 24].

Chronic nicotine use modifies multiple structures in the endogenous reward system of the brain [25, 26]. The pleasant and remarkably reinforcing experience of nicotine administration is associated with excessive neurochemical activity in limbic and paralimbic regions of the brain, particularly the ventral tegmental area, nucleus accumbens, and amygdala. In addition, conditioning links previously neutral stimuli with this reinforcing experience creating powerful cues that trigger increases in incentive salience. Over time, this system becomes dysregulated and associated with increased impulsive use of nicotine and decreased sensitivity to natural rewards (e.g., food, water, sex, nurturing) as well as significant executive function neural processing deficits [20, 23, 24, 27].

The Competing Neurobehavioral Decision Systems (CNDS) Model is a well-established dual-systems guide to conceptualizing the psychological processes involved with self-control (e.g., healthy, prudent, far-sighted decisions) in the context of immediately rewarding and less healthy choices (e.g., smoking) [28–30]. The CNDS model posits that decisions are driven by the interaction between two functional neurobiological networks: The executive function network, embodied in the PFC; and the impulsive network, embodied in the limbic and paralimbic regions of the brain [31–35]. Numerous behavioral, neuroimaging, and other studies support this contention [31–34, 36]. Greater PFC activity is linked with a higher likelihood of choosing more prudent, larger later rewards, even in the context of temptation [37]. Direct and indirect manipulation of the PFC appears to alter impulsive decision-making [30].

Among humans and animals, the value of reinforcers is discounted as a function of the time to receipt [38]. Most of us prefer immediate reinforcement but we are willing to wait for larger rewards to varying degrees [39]. The degree to which one discounts, or de-values, delayed reinforcers is called the delay discounting rate [40–43]. Discounting has been applied to a variety of commodities (i.e., money, health, cigarettes, drugs, etc.), magnitudes ($5, $100, $1000, etc.), and signs (i.e., gains, losses) [44]. Importantly, smokers consistently demonstrate higher discounting rates than non-smokers [44–49] and higher discounting rates among smokers are associated with a greater propensity for relapse [34, 50–54]. Discounting rates also reliably decrease with effective addictions treatment [35, 52, 53]. Anatomical and functional evidence indicates that delay discounting rates are associated with relative activity levels in two frontal-striatal neural circuits that parallel the functional neurobiological networks in the CNDS [31, 55, 56]. Imaging studies show that higher discounting rates are associated with decreased activity in the PFC relative to limbic/paralimbic activity [32, 56]. Delay discounting rate is now considered a biological marker for the relative functioning of the two networks described in the CNDS model [28, 30, 35, 57].

Repetitive transcranial magnetic stimulation (rTMS) is a non-invasive brain stimulation technique that selectively modulates neuronal activity by generating electrical currents produced by an electromagnetic coil [58–62]. When applied repetitively in consecutive pulses, rTMS can have a long-term impact on neural circuits [63]. High Frequency (HF) rTMS (> 1 Hz) of the PFC increases regional cerebral blood flow and cortical excitability and improves cognitive function, attention, learning, and memory [64–69]. rTMS has been FDA approved for the treatment of medication resistant unipolar major depressive disorder since 2008 [70]. Neuromodulation of the dlPFC with rTMS affects discounting rates [71, 72], and other types of impulsive and risky decision-making [19, 73–76]. In a preliminary study, 1 session (900 pulses) of HF rTMS (10 or 20 Hz) of the left dlPFC decreased delay discounting rates with 20 Hz rTMS demonstrating significantly larger effects on delay discounting rates than 10 Hz [10].

HF rTMS has demonstrated some potential for reducing cigarette consumption and craving in multiple studies [77–79]. Inconsistent findings among these studies is likely associated with variability in frequency (10-20 Hz), variability in the number of stimulation sessions (range 1–10), a lack of behavioral treatment components, variability in motivation to quit among participants, variability in stimulation site targeting method, variability in the assessment of craving, variability in procedures, and variability in the use of well-established abstinence outcome measures. HF rTMS of the PFC also has been shown to improve learning, memory, attention, and working memory as well as increase regional cerebral blood flow in addition to improving connectivity in the executive function network and enhancing working memory performance [64, 68, 80–82]. Proposed mechanisms of change include increasing long-term potentiation (LTP) and enhancing fronto-striatal pathway connectivity and dopamine function [83, 84]. LTP is one of several mechanisms involved with increasing synaptic plasticity by enhancing neuronal signal transmission [60, 66, 85]. LTP, through modifications of synaptic strength, appears to be one of the major cellular mechanisms that underlie learning and memory [86]. Finally, a systematic review of rTMS depression treatment studies suggests that rTMS of the PFC might be a promising technique for cognitive enhancement [87].

Many of the cognitive processes that comprise executive function are mediated by the dlPFC and are also important to cognitive behavioral skill acquisition. A cognitive-behavioral approach to the treatment for tobacco dependence promotes understanding of the cue-urge-response decision-making cycle, awareness of a multitude of conditioned internal and external cues encountered in daily life, and the development of choice options and strategies to manage incentive salience [88, 89]. Across the treatments of disorders, cognitive-behavioral treatment incorporates learning elements of self-control over one’s thoughts, behaviors, and feelings as reactions and emotions are monitored and thoughts are restructured (cognitive restructuring) as well as learning to activate particular behaviors in a range of contexts through anticipation of high-risk situations and planning (behavioral activation) [88, 90]. These acquired skills include self-monitoring, impulse control, stimulus control, stress management, and problem solving, etc. Cognitive-behavioral approaches can be delivered in varying intensities from self-help to intensive group or individual treatment, but all evidence-based “counseling” interventions for tobacco dependence are based on cognitive-behavioral principles [88, 89, 91] so we have included a cognitive-behavioral treatment in the current study.

Combining rTMS with cognitive-behavioral treatment has been shown to be feasible and therapeutically promising in the treatment of major depression [91], post-traumatic stress disorder [92], cognitive rehabilitation [93], and relapse to smoking [94]. The effects of HF rTMS of the PFC on learning, memory, and attention suggests that combining HF rTMS of the dlPFC with cognitive-behavioral interventions might facilitate the acquisition of cognitive-behavioral skills, enhance the overall effectiveness of the cognitive-behavioral intervention, and ultimately contribute to improved abstinence outcomes.

To address these limitations, we conducted a feasibility study that combined 8 sessions (900 pulses each session) of 20 Hz rTMS of the left dlPFC with an evidence-based cognitive-behavioral self-help intervention in a double-blind randomized sham-controlled trial with treatment-seeking smokers (*n* = 29) and multiple well-established tolerability and abstinence measures. The findings showed potential efficacy. It was found to be feasible and well-tolerated, to significantly decrease discounting rates, and to improve multiple abstinence outcomes [94]. In our feasibility study, we combined 8 sessions of 20 Hz rTMS of the left dlPFC with the a series of 8 evidence-based self-help booklets [94]. These 8 booklets, called Forever Free®, include multiple, practical cognitive-behavioral skills including understanding and intervening with the cue-urge-response cycle and engaging in cognitive restructuring and behavioral activation [95–97]. Treatment-seeking smokers (*n* = 29) who read one new booklet in order per session during the 8 stimulation sessions, were encouraged to continue reading outside sessions, and were followed for 12 weeks. Outcomes included latency to relapse, delay discounting rates, and 12-week, 7-day point prevalence abstinence rates. The mean latency to relapse for active rTMS was 45.19 (SD 9.42) days and sham 20.46 (SD 7.46) days. The median latency to relapse for active rTMS was 33.50 (IQR 7–85) and sham 8.00 (IQR 2 to 37) days. Active rTMS reduced the relative risk of relapse 3-fold (RR 0.29, CI 0.10–0.76, Likelihood ratio χ^2^ with 1 df = 6.40, *p* = .01). Active stimulation also decreased delay discounting of $100 (F (1, 25.3694) = 4.14, *p* = .05) and $1000 (F (1, 25.169) = 8.42, *p* < .01), increased 12-week abstinence rates (active 50% vs. sham 15.4%, X^2^ (df = 1) = 3.80, p = .05), and increased uptake of the materials. Side effects included headache and stiffness, but were minimal, resolved quickly, and were well-tolerated. Participants were engaged throughout treatment; 69% completed all 8 sessions with minimal compensation ($10 per session) and limited appointment availability due to staffing and access to the machine. These findings indicate that this approach has potential long-term efficacy, but more research is needed to determine the optimal dosing regimen to prepare for future efficacy testing.

While the premise and the preliminary evidence for this approach are compelling, an optimal dosing strategy must be determined before a Phase III trial with long-term efficacy outcomes can be conducted [98]. Dose determination studies are often carried out in Phase II of the development of an intervention to ensure that efficacy testing is conducted using the most promising dosing regimens [98]. Because the doses being tested in this protocol have already been found to be safe in many other studies [99–102], safety elements will be monitored, but safety is not the primary focus of this study. We follow a typical plan for a dose determination study by including a sham comparison and a range of lower to higher dosing regimens in an innovative, parsimonious factorial design [98].

The goal of this study is to determine a dosing strategy for 20 Hz rTMS that will produce the best long-term abstinence outcomes with the fewest undesirable effects. The aims of this study are to examine the effects of stimulation duration (stimulation days) and intensity (pulses per day) on outcomes among smokers (*n* = 258) who are motivated to quit smoking, and to identify the most promising dosing strategy by balancing effect sizes and undesirable effects. The study outcomes include latency to relapse, point prevalence abstinence rates, delay discounting rates, the acquisition of cognitive-behavioral skills, and potential undesirable effects, such as non-compliance and excessive participant burden. The findings will characterize the nature of the rTMS dose-response relations among two rTMS dosing parameters (e.g., duration and intensity). Our hypotheses are that the duration and intensity will be positively associated with effect size for latency to relapse, abstinence rates, delay discounting rates, and cognitive-behavioral skills acquisition and that the highest duration and intensity doses will be associated with greater levels of undesirable effects (e.g., noncompliance due to participant burden).

## Methods

**Fig. 1:**
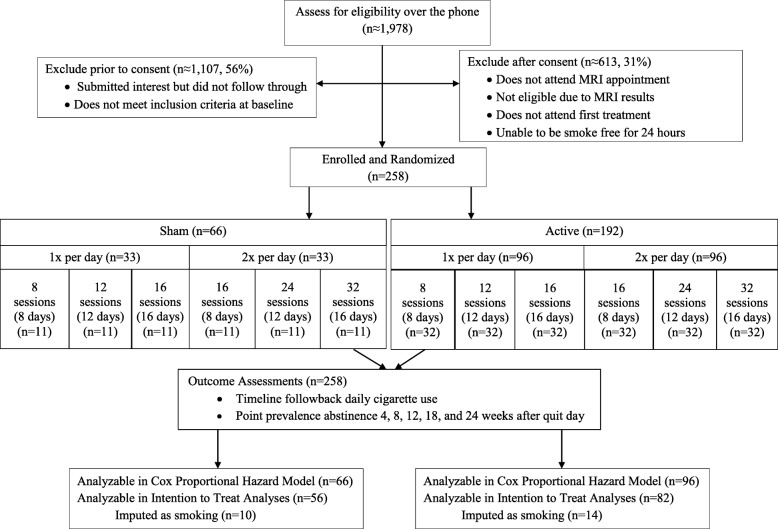
Study flow presenting anticipated number of participants at each milestone

This study is a fully crossed, double-blind, sham-controlled, 3x2x2 randomized factorial design. This factorial design is intended to screen duration and intensity components simultaneously to identify the most promising combination for an optimal dosing intervention for efficacy testing. The factors are duration (stimulation days: 8, 12, and 16), intensity (900 or 1800 pulses per day delivered in 1 and 2 sessions respectively), and a sham control for each condition (see Fig. 1). This study was submitted to, and approved by, Roswell Park Comprehensive Cancer Center’s (RPCCC) Institutional Review Board (IRB); all protocol modifications are sent to RPCCC’s IRB as well. Prior to completing any study-related procedures, participants will sign a written informed consent form. Participants in all conditions will be asked to complete 4 days of stimulation in a 7-day period and those assigned to conditions with 2 sessions per day will have sessions separated by at least 2 h. The days of stimulation must be completed within a specific timeframe (i.e., 8 days of stimulation within 14 days, 12 within 21 days, and 16 within 28 days). The sham control allows for a comparison group to calculate effect sizes while accounting for non-specific treatment-related effects. Participants are followed and assessed every 2 weeks for 24 weeks after initiating rTMS sessions. All in-person research activities will take place at RPCC’s campus, inside the Prevention Center building. The Prevention Center has its own parking and is well-suited for use as a research facility.

### Power analysis/sample size justification

Preliminary evidence suggests a mean of 20 days to relapse in the sham groups (*n* = 11). With *n* = 32 in the active groups and a common standard deviation of 20 × 0.40 = 8.0 across cells, this design has 80% power to detect a main effect of at least 4 days to relapse in a dichotomous factor (Cohen’s d = .50; 2-sided α = 0.05, SD = 8.0). With exponentially distributed days to relapse, a 4-day change in the mean equates to a detectable hazard ratio of 0.85 or less, in favor of the active condition.

Using the preliminary study as a guide, assuming days to relapse are exponentially distributed, the corresponding hazard ratio (active vs. sham) is between 0.24 and 0.45, based on means. This presents a relatively small-medium effect size but significant in the context of a study with *n* = 29 participants [103]. The observed days to relapse had an estimated coefficient of variation = 0.40. Preliminary evidence suggests a mean = 20 days to relapse in the sham groups (*n* = 11). The decision to include a control group improves the internal validity and confidence in effect attribution [98]. Because control groups impact feasibility (e.g., cost and size), we chose a factorial design in which two dosing components are simultaneously examined and reducing the size of the control group to the number needed to maintain precision, power, and the anticipated effect size. We estimate that we will be able to maintain estimated power and precision to detect the anticipated effect size with *n* = 11 in the sham control conditions (34% of the n for the active condition), for a total sample size of *n* = 258. Preliminary studies suggest that 14% of those we recruit will not attend any stimulation sessions and we plan to recruit *n* = 300 as a result.

### Participants

This study employs direct, community-based efforts focused on social marketing methods and small media (e.g., flyers in local businesses, advertisements in free local newspapers, talks with community groups). We aim to enroll *n* = 258 participants who receive at least one rTMS session. We anticipate that this study will enroll 50% women and 30% Black or African American smokers. Women and Black smokers make up a smaller proportion of the smoking population than White men, but women and Black smokers tend to be under-represented in many clinical trials and suffer disproportionately from tobacco-related disease. The population in Erie county, the county in which Roswell Park Cancer Institute is located, is about 14% Black [104].

To be included in this study, participants must meet the following criteria: be a healthy, right-handed adult, age 18–65 years old, negative urine drug and pregnancy test at the in-person screening assessment, able to read at the 8th grade level, pass the Transcranial Magnetic Stimulation Adult Safety and Screening Questionnaire [105], smoke between 5 and 25 cigarettes per day, and intend to quit smoking in the next 30 days. Participants with the following characteristics will be excluded: personal history of epilepsy; anticonvulsant medication use; current complications from head injury; history of aneurysm, stroke, or cranial neurosurgery; active diagnosis of migraine headaches, major depressive disorder, bipolar disorder, or schizophrenia-spectrum disorder; clinically significant findings on a magnetic resonance imaging (MRI; e.g., tumor, aneurysm, ischemic changes, etc.); metal implants or neurostimulators in the head, neck, or cochlea; a pacemaker; currently taking medications that lower seizure threshold or for tobacco cessation; are pregnant or planning to become pregnant in the next 6 months; currently use forms of tobacco other than cigarettes; score above 48.3 on the Claustrophobia Questionnaire at the baseline assessment (to ensure participants can undergo the MRI of the head); or are unwilling or unable to follow protocol requirements.

#### Equipment

Stimulation will be delivered with the Magstim Super RAPID^2^ PLUS1 System with Magstim 70 mm Double Air Film Active and Sham Figure of 8 Coils to deliver active and sham rTMS. Focal electrical stimulation will be delivered with a DS3 Isolated Stimulator (Digitimer Ltd., Welwyn Garden City, Herforshire, U.K.). Electrical pulses will be triggered by the TMS controller so that each pulse coincides precisely with the clicking sounds of the sham coil. The Brainsight for TMS Neuro-navigational system (Rouge Research, Inc.) will be used for precise placement of rTMS coils.

#### Procedures

Participants undergo extensive screening over the telephone prior to being invited to an in-person interview during which urine drug and pregnancy tests are administered. After providing informed consent, participants are administered a baseline assessment and undergo an MRI of the head (1.5 or 3 Tesla, no contrast, slice spacing 1x1x1mm), which is read by a radiologist. If the images reflect no contraindications for participation, participants are randomized and scheduled for the quit date, quit date counseling, and their regimen of rTMS sessions. See Table 1 for a summary of the study measures. The average time from baseline to first rTMS session is 30 days (SD = 20.4).

**Table 1:** Study measures

	Phone Screening	In-Person Interview	Baseline Assessment	1st TMS	All other TMS sessions	Outcome Assessments
Race, ethnicity, age	X					
Medication list (self-report)	X	X		X		
TMS Safety and Screening [105]	X	X				
Urine drug test		X				
Urine pregnancy test		X				
Claustrophobia questionnaire [106]		X				
MRI (Sagittal T1 Axial 3-D SPGR)			X			
Complete demographic information			X			X
Carbon monoxide level			X	X		X
5-Trial adjusting delay discounting task ($100, $1000) [107]			X			X
MacArthur scale of subjective social status [108]			X			X
Motivation to quit^a^			X	X	X	X
Self-efficacy to quit^a^			X	X	X	X
Treatment engagement^a^			X	X	X	X
Barratt impulsiveness scale-11 [109]			X			X
Time perspective questionnaire [110]			X			X
Perceived stress scale – 4 [111]			X	X	X	X
Positive and negative affect scale [112]			X	X	X	X
Center for epidemiologic studies depression scale [113]			X	X	X	X
State-trait anxiety inventory [114]			X	X	X	X
Cognitive-behavioral therapy skills questionnaire [115]			X			X
Perceived research burden) [116].			X			X
Side effects				X	X	
Booklet content tracking				X	X	X
Blinding questions (participant and staff) [117].				X	X	
Timeline follow-back daily cigarette use [118]						X

^a^Measured on a scale of 0–10 where 0 = not at all and 10 = the most possible

Participants receive reminder calls over the phone prior to the baseline, MRI, and all outcome assessments. Prior to the first rTMS session, quit counseling occurs over the phone and provides another opportunity to remind them of their appointments. In person, at the first rTMS session, study staff provide participants with a calendar showing all study visits. Study staff reiterate the importance of attending all rTMS sessions and calling if they are experiencing any study-related difficulties, including attending sessions. For the duration of the trial, participants are asked to refrain from the use of any smoking cessation aids, including nicotine replacement therapy in all forms as well as bupropion and varenicline.

### Stimulation site targeting

The dlPFC is a functional node in the PFC that lies in the middle of the frontal gyrus in the lateral part of Brodmann’s area 9 near 46 [119]. Prior to undergoing the MRI, we use the extended International 10–10 scalp electrode system to place a vitamin E capsule at the AF3 electrode position on the head to establish a fiducial marker for the stimulation site. The dlPFC is located between the F3 and the AF3 electrode positions [120]. The 10–10 system accounts for the curvilinear nature of the head, variability in head size, and some inter-session variability associated with other approaches [120]. The cognitive functions of interest are in the anterior region of the dlPFC [119] suggesting that the AF3 electrode position is the ideal site of stimulation. These procedures for achieving precision are highly consistent with current practice and recommendations [120, 121]. When the MRI of the head is uploaded into the Brainsight (Rogue Research, Inc) neuro-navigation system the fiducial marker is apparent on the image.

### Randomization

Random sequence allocation software is used to assign participants to treatment conditions which are concealed via password protected files until intervention group is assigned. To reduce inter-group variability, we use permuted block, stratified randomization by dependence level using the Fagerstrom test for nicotine dependence (FTND) score (low < 4, high ≥4). Nicotine dependence level significantly influences risk of relapse [122] and influenced outcomes in the feasibility study [94] and a pooled analysis of varenicline studies [123]. Project coordinators are the only individuals on the team who have access to the random assignment of participants. All other study and investigative team members, including technicians who administer rTMS and interviewers who assess outcomes, are blind to study assignment. Breaking the blinding code should only occur in situations when knowledge of the treatment is essential for participant care (e.g., due to severe adverse events (SAEs)). If unblinding is necessary, the already-unblinded project coordinator should work directly with the study physician rather than unblinding team members. Unblinding should not necessarily be a reason for discontinuation.

### Quit Day

The quit day is the day immediately prior to the first rTMS session. Participants are provided with psychoeducational materials and 30 min of brief structured counseling over the telephone at least 3 days prior to the quit attempt to help them achieve the initial 24 h of abstinence. Participants must abstain from smoking for at least 24 h as evidenced by an expired breath carbon monoxide level 10 ppm or less immediately prior to the first stimulation session [122]. Expired breath Carbon Monoxide (CO) levels of 8-10 ppm are recommended by the SRNT Subcommittee on Biochemical Verification as a cut-off for abstinence [122]. The half-life of CO varies by activity level, but can be as long as 8 h [122]. We use this cut-off for initial abstinence because higher CO levels, such as 40-50 ppm, are unlikely to reach lower than 10 ppm within 24 h among sedentary smokers who might have compromised respiratory systems [122]. Participants with expired breath CO levels greater than 10 ppm are encouraged to try again but do not initiate stimulation until they achieve 24 h of abstinence. Although 24-h abstinence is required for the first session to establish a valid quit attempt, abstinence is not required for receiving the remaining rTMS sessions.

### Preparation for rTMS

All participants are prepared identically. Individuals’ brains have varying levels of sensitivity to TMS pulses due to a variety of factors (thickness of skin and skull bone, space between scalp and brain tissue, etc.). The Motor Threshold (MT) is a well-established method of standardizing the stimulator impact on brain tissue. While seated in the stimulation chair, 3 electrodes are placed over the abductor pollicis brevis muscle (APB) on the hand. The MT is defined as the minimum stimulation intensity required to elicit a motor evoked potential of 50 μV from the APB in 3 of 6 trials. Once participants’ MT is determined, the stimulator is set to deliver stimulation at 110% of the MT. Once the MT has been determined, the rTMS technician and the participant leave the room and the project coordinator attaches either the active or sham coil as per random assignment and documents and double-checks accordingly. Once the appropriate coil is attached, the coordinator invites the technician and the participant to re-enter the room and preparations continue. The active and the sham coil are identical in appearance and set-up. Neither the participant nor the technician delivering stimulation will be able to tell a difference.

Prior to stimulation, conductive skin preparation gel is placed on two rectangular, carbon-impregnated rubber electrodes (4x5cm). The electrodes are placed firmly over the left frontalis muscle about 1 cm above the eyebrow underneath the headband that holds the Brainsight reflective tracking balls. The first electrode is aligned above the medial side of the eyebrow and the second aligned with the lateral aspect of the left eye. These electrodes deliver focal electrical stimulation in timing with the rTMS pulses as the sham technique but are not activated during the active rTMS condition.

### rTMS sessions

The Brainsight neuro-navigation system is used to control the angle and distance of the coil relative to the curvilinear surface of brain so that proper positioning is achieved, maintained, and replicated across sessions. Each session provides 900 pulses of 20 Hz rTMS of the left dlPFC at 110% of the MT. Pulses are delivered in 45 20-pulse trains of 1 s duration with an inter-train interval of 20 s. The actual stimulation time is 16 min. Participants read the 8 booklets, in order, during the first 8 days of stimulation. The booklets are added one-by-one to a packet which includes all the materials necessary for tracking content exposure. Participants take the packet home with them after sessions. Following each session, participants complete a post-session assessment.

### Outcome assessment

Participants are followed for 24 weeks after initiating rTMS. Number of cigarettes smoked per day is assessed every two weeks by telephone with the Timeline Follow Back (TLFB) procedure [124]. In-person outcome assessments are conducted 4, 8, 12, 18, and 24 weeks after first rTMS session. CO in exhaled breath is used to biochemically validate abstinence during in-person outcome assessment visits. CO levels of ≤5 ppm will be considered abstinent from smoking [122]. All participants who begin rTMS are invited to complete their OAs.

### Discontinuing participants

Participants may choose to end study involvement at any time and will not be replaced after starting their scheduled rTMS sessions. Any missed sessions are noted and participants are welcome to continue their treatment as scheduled (i.e., as they would had the sessions not been missed). We are unable to reschedule any rTMS sessions that fall outside of the 4-sessions in 7-days window.

#### Data analysis plan

Data will be analyzed at the end of the trial by the authors, including a project coordinator who is unblinded to study condition. With its wealth of resources, including biostatisticians, RPCC is well suited to assisting with any statistical concerns and a Data Monitoring Committee was not deemed necessary. In the feasibility study, the median and mean latency to relapse was 33.5 days and 45.2 days in the active condition, and 8 days and 20.5 days in the sham condition (RR 0.29, CI 0.10–0.76, Likelihood ratio χ^2^ with 1 df = 6.40, *p* = .01). Assuming days to relapse are exponentially distributed, the corresponding hazard ratio (active vs. sham) in the feasibility study was between 0.24 (based on medians) and 0.45 (based on means), a small to medium effect size but significant in the context of a study with *n* = 29 participants [103], and the observed days to relapse had an estimated coefficient of variation = 0.40. This suggests a mean of 20 days to relapse in the sham groups (*n* = 11) in that study. With *n* = 32 in the active groups and a common standard deviation of 20 × 0.40 = 8.0 across cells, this design has 80% power to detect a main effect of at least 4 days to relapse in a dichotomous factor (Cohen’s d = .50) (2-sided alpha = 0.05, SD = 8.0). With exponentially distributed days to relapse, a 4-day change in the mean equates to a detectable hazard ratio of 0.85 or less, in favor of the active condition. Please note, the factorial design reflected in Fig. 1 should not be considered a multiple arm randomized control trial (RCT) [125, 126]. Following recommendations from Collins et al., the sample size was estimated using smallest estimated effect size derived from the feasibility study (8 daily sessions in 14 days) [94, 125, 126].

Data is stored securely at the study site using both electronic and paper sources. Participant’s information will not be released outside of the study except as required for monitoring by the NIH or RPCC’s Clinical Research Services (CRS) department. All rTMS-related data is stored on paper source forms and is manually entered into SPSS using double data entry to ensure data quality. The battery of psychological assessments at baseline and outcome visits is stored electronically and automatically scored. Assessments are listed in full in Table 1 and references are provided showing reliability and validity for each measure. Scoring was coded and tested prior to the first participant and will be scored as a secondary check using SPSS syntax after study completion. All data exists on Roswell Park’s secure system, protected by the campus’ firewalls. The project coordinator will oversee intra-study data sharing with the guidance of the PI. To maintain confidentiality, all identifying information is blinded in the final dataset and the dataset is not shared with anyone outside the study team. Participation is monitored via status notification updates submitted to RPCC’s CRS. The CRS department is responsible for trial auditing at their discretion and the study team performs data quality control checks weekly. Results will not be communicated to participants directly but will be posted on ClinicalTrials.gov and published in a relevant journal agreed upon by all authors.

Prior to hypothesis testing, descriptive analyses will be conducted on all measures using appropriate summary statistics (i.e., mean and standard deviations, medians and interquartile range, and proportions). We anticipate 10–15% of participants will be lost to follow-up (LTF) after the first stimulation session. Initial analyses will examine treatment adherence and associations with missing data to determine whether missing data and censoring are non-informative (i.e., unrelated to the study). Sensitivity analyses will examine assumptions garnered from initial analyses and LTF will be examined as a study endpoint. Missing point prevalence outcome data will be addressed by imputing continued smoking for those LTF (i.e., intention to treat) and supplemented with multiple imputation analyses. All multivariate models will be computed using effect coding. Effect coding allows direct, independent estimation of main and interaction effects with 95% confidence intervals from a single model [127, 128]. Analyses will be conducted with and without adjusting for biologic variables (age, sex/gender) and race. All multivariate models will be specified to include effect-coded covariates for the duration (3 levels, ref.: 8 days), the intensity (ref: 1x per day) and the activity type (active, ref.: sham) and interactions to the 3rd order adjusting for biologically relevant covariates including the stratification factor. Multivariable adjustment generally improves the efficiency of analyses and avoids estimation bias from covariate imbalance. We will report outcomes appropriately quantified for the type of model used including hazard ratios, odds ratios, 95% confidence intervals, and *p*-values.

### Aim one

To examine the effects of stimulation duration and intensity on latency to relapse, point prevalence abstinence rates, delay discounting rates, cognitive-behavioral skills acquisition, and potential undesirable effects (e.g., non-compliance, participant burden, side effects) among smokers (*n* = 258) motivated to quit. *Hypothesis 1: Duration and intensity will be positively associated with effect size for latency to relapse, abstinence rates, delay discounting rates, and cognitive-behavioral skills acquisition*. We will use Cox proportional-hazards (CPH) models to examine latency to relapse outcomes. CPH models are appropriate for time-to-event data that include censored values [129, 130]. Data from participants who relapse are directly observed (i.e. not censored). Data from participants who are LTF or who do not relapse are censored on their date of last follow-up [131]. Ties in failure times will be handled using methods developed by Efron [132]. We will use logistic regression models to examine binary 12 and 24-week point prevalence abstinence. We will use repeated measures Generalized Linear models to examine delay discounting and cognitive-behavioral skills acquisition. *Hypothesis 2: The highest duration and intensity doses will be associated with greater levels of rTMS noncompliance due to subject burden.* We will examine the undesirable effects of duration and intensity using a repeated measures Generalized Linear model.

### Aim two

We will identify the most promising dosing regimen by systematically balancing efficacy and undesirable effects. Non-compliance is a primary focus of these analyses because noncompliance (i.e., non-adherence to session attendance) often emerges from multiple undesirable effects, including participant burden and side effects. To ensure the feasibility of a dosing regimen for efficacy testing, the proportion of participants who complete the regimen should be a least 45% [133, 134]. Based on preliminary work [10, 94], we expect at least 75% of participants to complete assigned stimulation sessions across conditions, with variability among conditions. Active dosing regimens with completion rates below 45% will be deemed intolerable and excluded from further consideration. For the remaining groups, selection of the most promising dosing regimen will be based on effect size and 95% confidence limit estimates, relative to appropriate controls and the impact of undesirable effects. Groups with overlapping confidence intervals will be considered equivalent, and the less burdensome treatment will be preferred.

### Adverse events

For this study, adverse events are monitored each visit by asking participants how they are feeling. Participants are encouraged to describe any differences they notice since the previous day and immediately following rTMS. All adverse events are recorded and those that meet the criteria for a serious adverse event (SAE) will be reported to CRS and the IRB. The investigators define SAE as any medical event believed to be causally linked to rTMS treatment and results in the following: a threat to the life of a participant; severe or permanent disability; or prolonged hospitalization. While the trial does not have ancillary care insurance, RPCC’s patient advocacy program functions as participant advocates as well and will be able to assist participants who experience adverse events related to study participation. Participants are advised of their services during the consenting process and would be reminded in the event of an adverse event. We have not experienced any SAEs as of the date of this publication. Unless the investigators feel rTMS or the study have caused them, SAEs that are noted after a participant completes or discontinues from the study will not be reported.

#### Preliminary participant characteristics

From October 2018 to January 2020, 503 people were screened for enrollment, *n* = 93 (18.5%) met initial eligibility requirements; of those, *n* = 59 (63%) initiated rTMS sessions and were fully enrolled. The participants recruited to date range in age from 20 to 65 (*M* = 50.2, SD = 10.6); 62.4% identify as female; and 70% identify as White or Caucasian, 23% as Black or African American, 2% as American Indian/Alaska Native, 1% as Asian or Pacific Islander, and 4% as other or multi-ethnic. Half of the participants are partnered. The average FTND score is 4.5 and the average number of cigarettes per day (CPD) is 14.57 (SD = 6.07). Of those that have completed their rTMS regimen (*n* = 58), 88% percent have completed all their assigned rTMS sessions across conditions to date.

## Discussion

This clinical trial integrates existing theoretical concepts, approaches, and methodologies from neuropsychology, behavioral economics, brain stimulation, clinical psychology, and the evidence-based treatment of tobacco dependence in the development of a promising and innovative approach to treat tobacco dependence. This study will establish an optimal dosing regimen for efficacy testing, the next essential next step in the development of this approach. The findings will significantly advance the research in the clinical application of rTMS as a treatment for tobacco dependence and perhaps other addictive disorders. In its early stages, this study is making progress in recruiting a relatively diverse group of participants. The percent of participants who complete their rTMS regimen as assigned across conditions is also promising and bodes well for future efficacy testing.

### Strengths and limitations

A significant strength of this study is the double-blinded, randomized, sham-controlled factorial design. Randomization and double blinding protect against bias and support the rigor of the approach while the factorial design parsimoniously screens for duration and intensity simultaneously. The sham control allows for a comparison group to calculate effect sizes while accounting for non-specific treatment-related effects. Another significant strength lies in the rigor and depth and breadth of the outcomes which assess a spectrum of the proposed cognitive and behavioral influences of rTMS. The TLFB assessment will provide a detailed continuous abstinence outcome appropriate for the CPH survival analysis, but will also provide information about the timing of relapses, the frequency of lapses, and whether participants recover from a relapse and regain continued abstinence for secondary analyses. The outcomes also include repeated assessment of delay discounting rates which will provide information about the pattern of delay discounting rates, an indicator of the relative balance of activity in the CNDS model across time. Important strengths also include the sample size, which provides enough power to confidently examine differences among all the outcome variables, and use of the CPH models which allow the inclusion of data from all participants who initiate treatment without imputation. We also see the intended representation of 50% women and 30% Black smokers in this study as a significant strength in terms of the generalizability of the findings.

This study has some potential limitations as well. This study is ambitious. While we have carefully evaluated our recruitment goals, our resources, our combined expertise, and the ideal study sample, and can demonstrate that the scope of this study is achievable in the 5-year timeframe, the recruitment goals remain ambitious and we estimate that it will require 75–85% capacity of our systems, which are open for scheduling from 8 am to 8 pm Monday-Friday. Thus, there is little leeway for accommodating unforeseen circumstances like equipment break-downs, staff illness/turnover, and weather emergencies. In addition, there is the chance that the treatment we ultimately develop might not be cost-effective. However, in order to calculate cost-effectiveness, we must first determine optional dosing and efficacy. This limitation, however, might be ameliorated in the future given the range of health care providers who are able to deliver rTMS has increased dramatically since 2013. In concert, we expect the cost to continue to decrease in the next several years. Once dosing and efficacy are determined, we can determine cost per quit and quality of life years saved to determine cost-effectiveness. Recent evidence suggests that rTMS treatment for depression is more cost-effective than anti-depressant therapy [135, 136]. Finally, the design relies on smokers to attain 24 h of abstinence prior to stimulation and this might be too difficult or too discouraging for some participants. While attrition prior to the first session of any treatment for tobacco dependence is common and in the feasibility study participants reported that scheduling and other issues caused non-attendance, we suspect that the inability to achieve 24 h of abstinence contributed to a lack of compliance in the feasibility study. For this study, we revised our approach to provide standardized counseling and assistance at least 3 days prior to the quit attempt to help participants plan for and be successful with their 24-h quit. As in the feasibility study, participants with expired breath CO levels > 10 ppm will be encouraged to try again, but will not initiate stimulation until they achieve ≤10 ppm. In defense of this requirement, which is intended to standardize the quit date and support a valid quit attempt, over half of smokers attempt to quit every year and the vast majority of these attempts last > 24 h [3, 137].

In conclusion, the findings from this study are expected to have a sustained and powerful influence on advancing this novel approach to treating tobacco dependence. This study is the next logical step in the development of a new approach for preventing relapse to smoking, which was previously found to be feasible and to have potential long-term efficacy. The findings will inform future research on clinical approaches that combine rTMS with other evidence-based treatments for tobacco dependence and perhaps other addictions.

## Data Availability

The datasets generated and/or analyzed during the current study are not publicly available due to the ongoing nature of the study but will be available from the corresponding author upon reasonable request.

## Abbreviations

APB: Abductor pollicis brevis
CNDS: Competing Neurobehavioral Decision Systems
CLQ: Claustrophobia questionnaire
CO: Carbon monoxide
CRS: Clinical research services
dlPFC: Dorsolateral prefrontal cortex
FTND: Fagerstrom test for nicotine dependence
HF: High frequency
IRB: Institutional Review Board
LTF: Lost to follow-up
LTP: Long-term potentiation
MT: Motor threshold
MRI: Magnetic resonance imaging
PFC: Prefrontal cortex
rTMS: Repetitive transcranial magnetic stimulation
SAE: Serious adverse event
